# Status of Hawksbill Turtle and Green Turtle in Negeri Sembilan, Malaysia

**DOI:** 10.21315/tlsr2024.35.2.3

**Published:** 2024-07-31

**Authors:** Sarahaizad Mohd Salleh, Shahrul Anuar Mohd Sah

**Affiliations:** 1 Environmental Science Centre, H10 Research Complex, Qatar University, P. O. Box 2713 – Doha, Qatar; 2 School of Biological Sciences, Universiti Sains Malaysia, 11800 USM Pulau Pinang, Malaysia

**Keywords:** Clutch Size, Green Turtle, Hawksbill, Nesting, Negeri Sembilan, Jumlah Telur, Penyu Agar, Penyu Karah, Sarang, Negeri Sembilan

## Abstract

This paper highlighted the annual distribution, seasonality and reproduction status of two species of sea turtles in Negeri Sembilan, Peninsular Malaysia between January 2016 and July 2020 (55 months). These data were officially provided by the Department of Fisheries Malaysia (Negeri Sembilan’s state), as a part of a conservation effort made by them to protect the endangered species, as the turtle’s population residing in Negeri Sembilan is one of the smallest in Malaysia. Current status shows that the hawksbill turtles, *Eretmochelys Imbricata* population in Negeri Sembilan is in stable decline with an average of <30 nests per year, and an average of <10 nests per year for the green turtle, *Chelonia mydas*. The overall sum was 122 nests collected for both species from 2016–2020. This paper updated the status of the hawksbill and green turtle after 30 years from the first study recorded by Mortimer *et al*. in 1993.

## Abstrak

Kertas kajian ini menitikberatkan dapatan taburan tahunan, musim dan kadar pembiakan untuk dua spesis penyu di Negeri Sembilan, Semenanjung Malaysia diantara bulan Januari 2016 sehingga Julai 2020 (55 bulan). Data ini mendapat kebenaran rasmi dari Jabatan Perikanan Malaysia (Negeri Sembilan) untuk diterbitkan, salah satu daripada usaha pemuliharaan dari Jabatan untuk menyelamatkan hidupan terancam, kerana populasi penyu di Negeri Sembilan adalah antara yang terkecil di Malaysia. Status terkini menunjukkan populasi penyu karah, *Eretmochelys Imbricata* di Negeri Sembilan berada pada tahap penurunan yang stabil dengan jumlah purata tahunan sebanyak <30 sarang, dan purata tahunan sebanyak <10 sarang bagi spesis penyu agar, *Chelonia mydas*. Jumlah keseluruhan pula adalah sebanyak 122 sarang daripada kedua-dua spesis ini dari tahun 2016–2020. Kertas kajian ini mengemaskinikan status penyu karah dan penyu agar selepas 30 tahun, dari kajian pertama yang direkodkan oleh Mortimer *et al*. pada tahun 1993.

**Highlights**

- The overall sum of nests was 104 for hawksbill turtle and 18 for green turtle during four years of observations (2016–2020).
- The hawksbill turtles population is in stable decline, with new evidence of green turtle nesting collect between 2016–2020. The annual range,<30 nests was almost the same with a slight increase in comparison with<25 nests annually in the the past three decades, starting from 1991/1992 in the state of Negeri Sembilan.
- Two species identified landing at Port Dickson beach are the green turtle, *Chelonia mydas* and the hawksbill turtle, *Eretmochelys imbricata*. Previously, there was no evidence of green turtle landing since 1993.

## INTRODUCTION

The global status of the hawksbill turtle, *Eretmochelys imbricata* is currently listed as a “Critically Endangered” species and “Endangered” species for the green turtle, *Chelonia mydas* (Mortimer & Donnelly 2008). Hawksbills have been recognised to be residing in Melaka (Mortimer *et al*. 1993), Sabah (Chan *et al*. 1999) with fragment distribution in Terengganu (Chan 2010; Chan 2013) and Negeri Sembilan (Mortimer *et al*. 1993). Negeri Sembilan is located on the west coast of Peninsular Malaysia, and the border with Melaka has been known as an important nesting ground for hawksbill turtles (Mortimer *et al*. 1993). Negeri Sembilan is famous as a tourist attraction spot (i.e., water sports, camping). Therefore, it is important to patrol the beaches and control human disturbance during the nesting season by the authorised person. Or else, human disturbance can result in false crawls and egg poaching, especially during public holidays, when human density is high.

**Figure 1:**
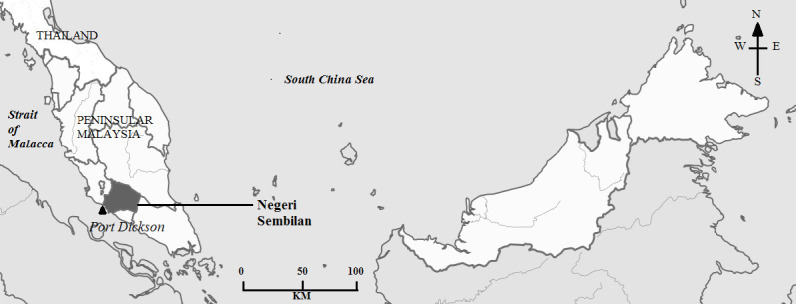
Map of Negeri Sembilan, located on the west coast of Peninsular Malaysia *Source*: created by GPS Visualizer

The objectives of the study are to observe the annual distribution, seasonality and reproduction of sea turtles from 2016 to 2019 in Negeri Sembilan, Peninsular Malaysia (Fig. 1). We also would like to confirm the current species landing, as previously by Mortimer *et al*. (1993) in their initial study that estimated >25 hawksbill turtles nesting per year in Negeri Sembilan, and no green turtles mentioned landing. To present the outcome, yearly nests data from January 2016 until June 2020 were retrieved from the Department of Fisheries Malaysia (DoF)with their approval to publish the research to the readers. Reproduction data of clutch sizes and annual hatchling success produced from the hatchery at Pusat Ikan Hiasan, Port Dickson, Negeri Sembilan was also an important study and will be presented in this paper. This paper highlighted the current nesting distribution and status for sea turtles in Negeri Sembilan, which is crucial in terms of conservation as sea turtles are declining globally due to human settlement, social activities and loss of nesting habitat.

## MATERIALS AND METHODS

### Nocturnal Surveys

Three beaches have been identified as nesting beaches in Negeri Sembilan: Port Dickson Beach – approximately 13 km, Pasir Panjang – approximately 3 km, and Tanjung Gemok – approximately 5 km (Jabatan Perikanan Malaysia 2016). The patroller walked slowly along the sandy beach (approximately 21 km stretch beach) using minimal light to verify any landing tracks. To avoid the disturbance, the nesting data were recorded and relocated after the turtle had finished nesting and returned to the sea. No flask camera was allowed during the nesting process. During the surveys, the patroller and researcher were advised to wear properly covered shoes, socks and dark-coloured shirts and trousers. As Negeri Sembilan holds a small population of sea turtles (Mortimer *et al*. 1993), 1–2 patrollers monitored the Port Dickson beach from 0800 to 0500 h. The standard guidelines for monitoring turtles and collecting turtle eggs can be referred in the updated manual book by Rusli *et al*. (2020). Yet, as Port Dickson is famous for tourist activity, visitors are advised to report immediately if encountered turtles landing, also strongly advised not to disturb the nesting activity at night. If the turtles had left the beach, nests were re-track by following the nesting track left by the female, and the patrollers will use metal rod to prick the sand to identify those eggs. Eggs were transported to the hatchery (50% shaded) namely Pusat Ikan Hiasan located at Port Dickson, Negeri Sembilan as soon as possible for incubation until the eggs hatched and those nests were patrolled daily by hired DoF personnel. The reproduction output, which is the number of hatched and unhatched eggs was counted. Hatchling success (%) was calculated according to the formula (Miller 1999; Hitchins *et al*. 2004; Zare *et al*. 2012):

The nest must be relocated to a much safer place as social activity is high and difficult to be controlled at Port Dickson beach. Information/nesting output such as the date of nesting, species and total eggs incubated were labeled at each nest. Nests were protected with squared netlon mesh that was placed around the nest to avoid predator attacks such as monitor lizards and crabs. Approximately after 40 to 45 days of incubation, an additional green wire-mesh fence (±70 cm diameter × ±150 cm length) was placed around the nest; buried at 15 cm depth to avoid smells of hatched eggs inside the nest that attracts land predators from digging the nest. In addition, avoiding hatchlings’ emergence that crawls by themselves making their journey to the sea. This standard method in terms of beach surveying, night observation and animal handling was performed according to the official guidelines by DoF and with staff guidance (Sukarno *et al*. 2007; Rusli *et al*. 2020). Studies were performed along the beach of Port Dickson and were authorised by the Director of the DoF and with their permission to conduct this research within the area. Port Dickson Beach was surveyed 2 to 3 times per week between 2100 h to 0500 h. Nests were also collected based on reports made by the public at certain remote beaches such as Tanjung Gemok and Pasir Panjang.

### Statistical Analysis

We used SPSS 17.0 version (Pallant 2002) and Microsoft Excel to analyse the result. One-way analysis of covariance (ANCOVA) was used to analyse the independent variables clutch sizes, and dependent variables consisting of months (January–December) through 2016–2020 (continuous covariates). The number of hatched eggs was used as a covariate in this analysis. A preliminary check was conducted before performing one-way ANCOVA to ensure that there was no violation of the assumption of normality, linearity, homogeneity of variance and reliable measurement of the covariate. A two-way analysis of variance (ANOVA) was performed to study the effect of two independent variables (months and years) on the distribution of clutch sizes (dependent variable). A Chi-squared test was used to analyse the uniformity of seasonality. Additionally, Poisson regression is used to predict a dependent variable that consists of count data given one or more independent variables. In this case, Poisson regression is used to predict the distribution of clutch sizes per month according to two species landing in Negeri Sembilan. The Kruskal-Wallis test (the nonparametric test) was used to test the significant differences between the groups of clutch sizes (0–50, 51–100, 101–150, 151–200 eggs). Lastly, a GPS Visualizer was used to create a map (Fig. 1).

## RESULTS

### Nesting Distribution

**Figure 2:**
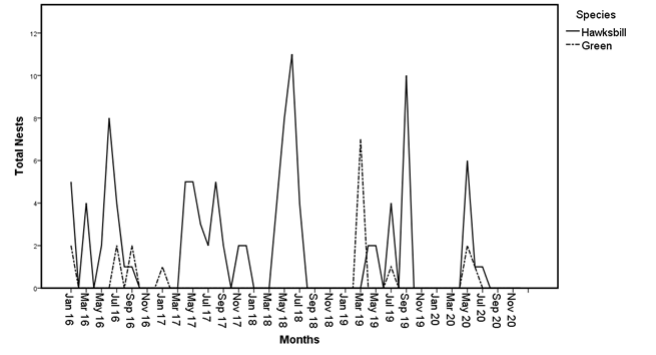
Bar graph of continuous nesting per month over 55 months, from 2016 to 2020.

**Figure 3:**
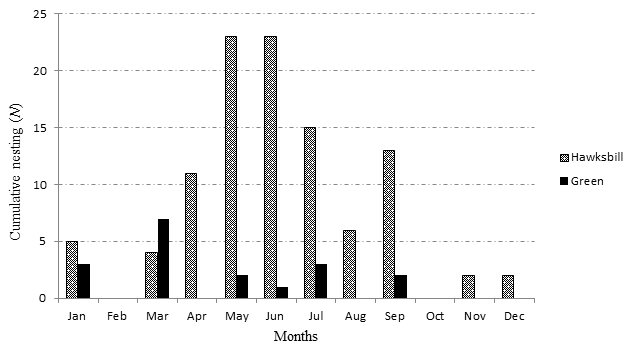
Cumulative nesting per month over five-years from 2016 to 2019 in Negeri Sembilan, Malaysia.

Two species identified landing at Port Dickson beach are the green turtle, *Chelonia mydas* and the hawksbill turtle, *Eretmochelys imbricata*. The annual range of hawksbill nests was 8–27 nests from the year 2016 to 2020 (Table 1). While for the green, nests annually ranged between 0–8 nests from the year 2016 to 2019. The overall sum of nests was 104 for hawksbill turtles and 18 for green turtles during four years of observations. Over the 55 months, from 2016 to 2020, the monthly number of hawksbill nests ranged from 0–11 (Fig. 2), and the figure explained the number of hawksbill nests each month had high variability across years. From the cumulative nesting, the peaks of the highest number of hawksbill nests generally occurred in May, June and July (Fig. 3). The highest peaks occurred in June 2016, June 2018 and May 2020, and somehow, the peak also observed occurred in September 2019 (Fig. 2). Both hawksbill and green show similar trends of nesting per month throughout the five seasons as there is no statistically significant association between both species, Chi-squared test, that χ^2^(11) = 0.000, *p* = 1.000 (Fig. 3). A similar trend of high variability was also observed for the green turtle nesting trend in Negeri Sembilan, with monthly nesting ranging from no nesting to the highest of seven nests that occurred in March 2019 (Fig. 2). From 2016 to 2020, green turtle landing and nesting only recorded in January, July and September in 2016, January 2017, March and July 2019, and May 2020.

### Clutch Sizes

The clutch sizes of hawksbill turtles ranged between 42–159 eggs (mean ± SD = 70.48 ± 19.56, median = 65.50, *N* = 104). While for the green turtles, clutch sizes ranged between 42–150 eggs (mean ± SD = 75.17 ± 26.59, median = 63.00, *N* = 18) during 2016–2019 observations. A two-way ANOVA was conducted to examine the effect of months and years on the distribution of clutch sizes. There was no statistically significant interaction between the effects of months and years on clutch size laid, *F* (14, 94) = 0.925, *p* > 0.05.

**Figure 4:**
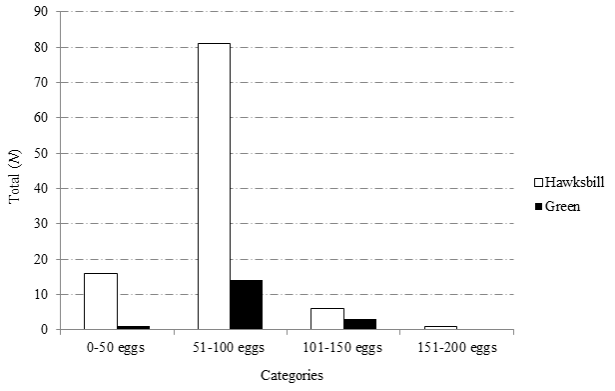
Clutch size distribution for the hawksbill turtle and the green turtle.

To test the differences in clutch size distribution over three categories for both species, Kruskal-Wallis test was used since these data were not normal according to the normality test (K-S = 0.137, df = 122, *P* < 0.001). Clutch sizes for both species were grouped into four categories (range of eggs laid) (Fig. 4) and mean rank shows non-uniformity distribution according to the Kruskal-Wallis test (χ^2^ = 63.71, df *=* 3, *P* < 0.001, *N =* 122). Fig. 4 shows that both the hawksbill and the green laid eggs the most at a range of 51–100 eggs (overall 77.9%) follow by the range of 0–50 eggs (13.9%) and 101–150 eggs (7.4%). Only one nest hawksbill was observed falls at the range of between 151–200 eggs.

**Figure 5:**
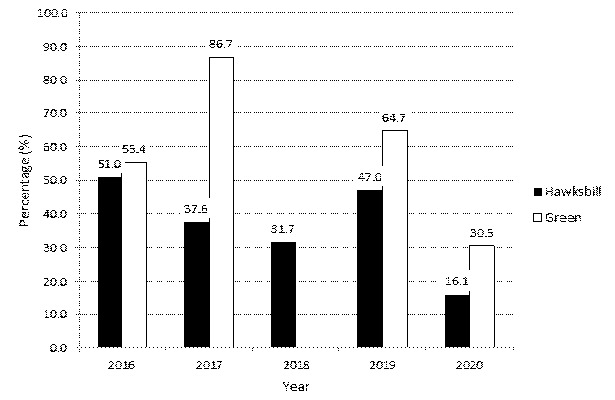
Annual mean percentage of hatching success.

**Figure 6:**
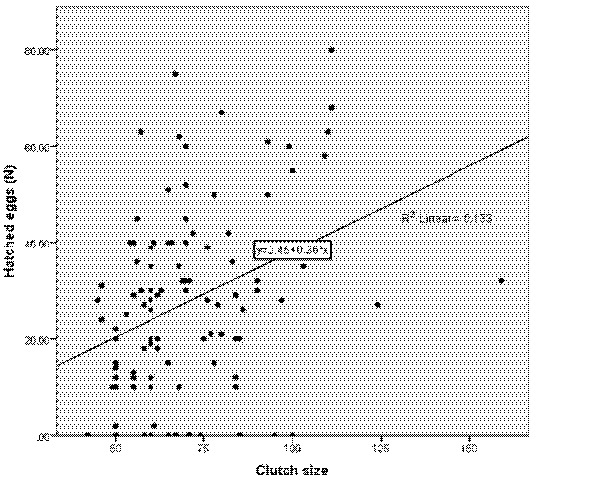
Linear regression analysis between clutch size and hatched eggs. 58

One-way ANCOVA was performed, and there was a significant interaction between clutch sizes and the number of hatched eggs, *F* (9, 93) = 2.176, *P* < 0.05, partial eta-squared = 0.2. There was a moderate relationship between clutch sizes and the number of hatched eggs across the months, as indicated by a partial eta-squared of 0.3 (Fig. 6). The annual mean hatching success ranged from 16.1% and 51.0% (overall mean ± SD = 36.7% ± 12.3) for the hawksbill, and between 30.5% and 86.7% (mean ± SD = 59.3% ± 20.2) for the green (Fig. 5).

## DISCUSSION

The latest nesting density was <30 hawksbill turtle nests that were found annually between 2016 and 2020, and all of them were collected at Port Dickson Beach. The annual range was almost the same with a slight increase in comparison with <25 nests annually as Mortimer *et al*. (1993) mentioned in the past three decades, starting from 1991/1992. in the state of Negeri Sembilan. In addition, evidence of <10 nests of green turtles being collected annually is a good sign of positive conservation. Previously, there was no evidence of green turtle landing as stated by the initial study by Mortimer *et al*. (1993). Since Negeri Sembilan is a border with Melaka, it was found that the trend of nesting where the peak occurred mostly in May, June and July in Negeri Sembilan is similar to the peaks in Melaka (Sarahaizad *et al*. 2018a). From this result, it is indicated that Negeri Sembilan is the second largest nesting rookery for hawksbill turtles in Peninsular Malaysia after Melaka (Mortimer *et al*. 1993; Sarahaizad *et al*. 2018a), followed by Redang Island, Terengganu with less than 0.3% hawksbill recorded annually (Chan 2010). It was observed that the mean and highest clutch size collected for hawksbill turtle in Negeri Sembilan was a bit smaller in comparison with clutch sizes in Melaka, where the highest mean was 123.5, and clutch sizes ranged between 9–212 eggs (Sarahaizad *et al*. 2018a).

In addition, with around 25–35 nests sum from the hawksbill and the green turtle species collected annually, Negeri Sembilan is probably the smallest nesting rookery for sea turtles in Peninsular Malaysia based on the comparison of annual nests collected at important rookeries in Penang island with 43–61 green turtle nests (Sarahaizad *et al*. 2020), Perak with 10–220 green turtle nests (Sarahaizad *et al*. 2018b), Melaka with 450–500 hawksbill turtle nests (Sarahaizad *et al*. 2018a) and Terengganu (Redang Island) with 221–687 green turtle nests (Chan 2010) and Setiu with 28–201 green turtle nests (Aini Hasanah *et al*. 2014).

In the current situation, the Negeri Sembilan government today agreed to gazette the law, turtle and Turtle Egg Fisheries Rules 1976 in the state to preserve adult turtles landing and nests, and to secure that the matter regarding turtle conservation was protected (*Utusan Malaysia* 2021). This law empowers the Fisheries Officer to carry out enforcement and issue licenses (*Utusan Malaysia* 2021) which will give positive feedback such as controlling beach activity, night disturbance, and egg poaching. As stated by the Chief Minister of the state, Datuk Seri Aminuddin Harun, the law is originally under the Fisheries Act 1963, and currently a subsidiary under the Fisheries Act 1985, and is indeed relevant to enforce in Negeri Sembilan. In addition, the implementation of the Movement Control Order (MCO) to curb the spread of the COVID-19 pandemic since 18 March 2020 showed a positive outcome that attracted sea turtles to land and lay eggs earlier than usual at Port Dickson Beach, Negeri Sembilan (*Harian Metro* 2020). The discovery of 118 and another 110 green turtle eggs landing at different times were shared and confirmed by the Negeri Sembilan Fisheries Department, and those eggs were relocated for incubation. These productive outcomes are believed to be caused by the decline in human density, resulting in less artificial light, especially from fishing and camping activities. In addition, less human activity also lessens ocean pollution as this beach is a famous tourist spot for fishing and camping. Debris such as plastic bottles, plastic bags, aluminum canes, glasses and styrofoam fragments are the common trash found on the beach and swiped to the ocean from camping and fishing activity, which could destroy the marine ecosystem.

The annual mean hatching success rate is a bit low for hawksbill turtles (<55%), and high for the green turtles (<90%). Eggs suffer a low hatch rate probably due to harsh handling during the transportation journey or long hours duration time of incubation at the natural nest, before being reburied and transferred the nest to the hatchery.

### Threats Currently Occur That Need To Be Seriously Overcome Towards Better Conservation In Negeri Sembilan

#### Human density problem

Port Dickson experiences a human density problem as the location is a famous tourist spot for Malaysians, which slightly impacts the landing of sea turtles. Other threats identified are continuous pollution problems which could affect the landing of turtles and destroy the marine habitat, beach, ecosystem, seagrasses, live coral reefs and fish population. “Kaedah-kaedah Perikanan Sungai 1976” was currently enforced in Negeri Sembilan (*Utusan Malaysia* 2021). This law involves the management and restriction of fishing activities in rivers, including lakes and swamps in Negeri Sembilan. This could give a positive impact in slightly controlling human density and human anthropogenic disturbance (camera flash, campfire) at Negeri Sembilan during nocturnal surveys by DoF patrollers. In addition, Fisheries Act 1985 and Fisheries Methods (Turtle and Eggs) 1989 were enforced to control the illegal smuggling of turtle eggs and banned the sale of eggs openly in the market (Abd-Rahim 2016) which could control the aggressiveness of eggs poaching due to the high in demand.

#### Active fisherman activity

Active fisherman activity at Port Dickson using trawl nets (*pukat tunda*) could cause accidental turtle capture, and the most recent report mentioned turtles drowning in traps in abandoned nests. This is among the problems faced by turtle conservation centers in most Malaysian states including the DoF team of Negeri Sembilan. There is a need to seriously spread information and educate fishermen on the prohibition of discarding abandoned nets into the sea, and leaving finer mesh to drift unattended as these could trap and drown the sea turtles. Currently, the Turtles Enactment 1951 (Amendment 1989) banned the of large mesh that exceeds 5 cm sunken gill nets to capture rays (Chan 2006). Based on the survey conducted in the Terengganu oceans in 1988, the drift nets with mesh sizes greater than 17.8 cm were capable of catching 16 turtles in one operation (Sukarno & Omar 1989). The study resulted in a nationwide ban in 1989 on the use of drift nets with mesh sizes greater than 25.4 cm (Yeo *et al*. 2007). Other efforts made to reduce the turtle’s accidental capture is the DoF has divided the fishing zone and division of operational vessels into four zones based on certain criteria and the performance of the fishing vessels (Yaakob *et al*. 2015). The first zone is Zone A, which covers operational areas less than 5 nautical miles from the shore. This zone is safe and suitable for traditional boats (sampans) and small fishing vessels with Gross Tonnage (GRT) between 0 to 19.9 GRT which the small fishing vessel not going to harm the sea turtles near the shores. The second zone is Zone B which covers areas between 5 to 12 nautical miles from the shore, and this zone is acceptable for medium size fishing vessels with GRT between 20 to 39.9 GRT which usually uses drag nets for fishing activity. Zone C covers fisheries operation areas from 12 to 30 nautical miles, which are suitable for trawlers and purse seiners. Lastly is Zone C2 which covers more than 30 nautical miles.

#### Water activity

We also suggest water sort activity management such as jet skis and speed boats must be operated farther from the shores (turtles often swim and mate near the shores) with a speed limit as there are few cases of turtles being hit by boat engines and cause carapace crack from the sharp blade. In addition, oil spills, noise pollution, and light pollution from project reclamation sites in Port Dickson need to be seriously mitigated. Consequential mitigation must be implemented by monitoring the nesting for every season to avoid a drastic decline in sea turtle landings. In this current situation, many nesting habitats in Malaysia have been lost due to land reclamation, pollution and beach-front development and alterations.

## CONCLUSION

This paper provides basic information on the latest current conservation implemented by DoF in Negeri Sembilan. The outcome is important for further protection as currently, the hawksbill turtles population is in stable decline, with new evidence of green turtle nesting collect between 2016–2020. Debris reduction, declining of human density and reduction of artificial light and ocean pollution due to pandemic COVID-19 has proven a positive outcome in attracting turtles to land. Therefore, regular beach cleaning activities and control of human social activities during nocturnal may help towards future conservation in gaining the attraction of sea turtles landing in Negeri Sembilan.

## Figures and Tables

**Table 1:** Nesting density, the sum of nesting, mean and median of clutch size, total eggs and survival hatchlings for hawksbill turtle in Negeri Sembilan, Malaysia.

Location	Species	Year	Total nests observed (%)	Sum of nests	Clutch size		Total eggs	Overall sum of eggs	Survival hatchlings

					Mean ± SD	Median			
Negeri Sembilan	Hawksbill	2016	25 (24.0)	104	82.4 ± 17.6	77.0	2,059	7,330	1,050
		2017	26 (25.0)		67.7 ± 14.4	64.0	1,761		663
		2018	27 (26.0)		65.7 ± 12.6	63.0	1,773		562
		2019	18 (17.3)		57.3 ± 9.4	53.0	1,031		485
		January–June 2020	8 (7.7)		88.3 ± 36.0	77. 5	706		114
	Green	2016	6 (33.3)	18	73.2 ± 20.1	73.0	439	1,353	243
		2017	1 (5.6)		60 ± 0.0	60.0	60		52
		2018	0 (0.0)		0 ± 0.0	0.0	0		0
		2019	8 (44.4)		67 ± 11.2	60.0	536		347
		January–June 2020	3 (16.7)		106 ± 40.6	116.0	318		97
	Total		122				8,683		3613

## References

[b1-tlsr-35-2-51] Abd-Rahim RI (2016). Akta kawalan lama wujud tetapi tidak dikuatkuasa. Berita Harian.

[b2-tlsr-35-2-51] Aini Hasanah AB, Nik F, Amiruddin A, Nurolhuda N (2014). Understanding nesting ecology and behaviour of green marine turtles at Setiu, Terengganu, Malaysia. Marine Ecology.

[b3-tlsr-35-2-51] Chan EH (2006). Marine turtle in Malaysia: On verge of extinction?. Aquatic Ecosystem Health and Management.

[b4-tlsr-35-2-51] Chan EH (2010). A 16-year record of green turtle and hawksbill turtle nesting activity at Chagar Hutang Turtle Sanctuary, Redang Island, Malaysia. Indian Ocean Turtle Newsletter.

[b5-tlsr-35-2-51] Chan EH (2013). A report on the first 16 years of a long-term marine turtle conservation project in Malaysia. Asian Journal of Conservation Biology.

[b6-tlsr-35-2-51] Chan EH, Joseph J, Liew HC (1999). A study of hawksbill turtle (*Eretmochelys imbricata*) of Pulau Gulisaan, Turtle Islands Park, Sabah, Malaysia. Sabah Park Natural Journal.

[b7-tlsr-35-2-51] Harian Metro (2020). PD yang sepi galak penyu bertelur.

[b8-tlsr-35-2-51] Hitchins PM, Bourquin O, Hitchins S (2004). Nesting success of hawksbill turtles (*Eretmochelys imbricata*) on Cousine Island, Seychelles. Journal of Zoology.

[b9-tlsr-35-2-51] Miller JD, Eckert KL, Bjorndal KA, Abreu-Grobois FA, Donnelly M (1999). Determining clutch size and hatching success. Research and management techniques for the conservation of sea turtles.

[b10-tlsr-35-2-51] Mortimer JA, Ahmad Z, Kaslan S (1993). The status of the hawksbill, *Eretmochelys imbricata* and green turtle, *Chelonia mydas* of Melaka and Negeri Sembilan. Malayan Nature Journal.

[b11-tlsr-35-2-51] Mortimer JA, Donnelly M (2008). Eretmochelys imbricata. IUCN 2010: IUCN Red List of Threatened Species Version 20101.

[b12-tlsr-35-2-51] Pallant J (2002). SPSS survival manual: A step by step guide to data analysis using SPSS for Windows (Version 12).

[b13-tlsr-35-2-51] Rusli MU, Sahibu A, Azlan NS, Luqman F, Emilia T (2020). Modul latihan pengurusan pemuliharan penyu (jurulatih) [Module].

[b14-tlsr-35-2-51] Sarahaizad MS, Nishizwa H, Mohd-Sah SA, Safri MF (2018a). Spatiotemporal preferences in nesting of the hawksbill turtle (*Eretmochelys imbricata*) in Melaka, Malaysia. Journal of the Marine Biological Association of the United Kingdom.

[b15-tlsr-35-2-51] Sarahaizad MS, Mohd-Sah SA, Khan Chowdhury AJ (2018b). Assessing nesting status of green turtles, *Chelonia mydas* in Perak, Malaysia. Tropical Life Sciences Research.

[b16-tlsr-35-2-51] Sarahaizad MS, Nishizawa H, Shahrul Anuar MS, Khan Chowdhury AJ (2020). Reproductive seasonality and environmental effects in green turtle (*Chelonia mydas*) nesting at Penang Island, Malaysia. Journal of the Marine Biological Association of the United Kingdom.

[b17-tlsr-35-2-51] Sukarno W, Omar H (1989). Kematian penyu disebabkan kegiatan menangkap ikan.

[b18-tlsr-35-2-51] Sukarno W, Mohamed-Ridzuan MA, Mohamad-Zabawi S, Mohd-Najib R, Abdul-Aziim MY, Mansor Y, Azwa AH, Farizan S, Mohd-Khalil-Khasah M, Robert LHF, Abd-Karim S, Zakaria S, Syed-Abdullah SAK, Zulkifli T, Wahidah MA, Abdul-Wahab A, Norul-Fahiezah S (2007). Prosedur piawaian pengurusan penyu Semenanjung Malaysia.

[b19-tlsr-35-2-51] Utusan Malaysia (2021). Kerajaan Negeri Sembilan warta undang-undang pemeliharaan penyu.

[b20-tlsr-35-2-51] Yaakob O, Hashim FE, Jalal MR, Mustapa MD (2015). Stability, seakeeping and safety assessment of small fishing boats operating in Southern coast of Peninsular Malaysia. Journal of Sustainability Science and Management.

[b21-tlsr-35-2-51] Yeo BH, Squires D, Ibrahim K, Gjertsen H, Syed Mohd Kamil SK, Zulkifli R, Groves T, Hong MC, Tan CH (2007). Fisher profiles and perceptions of sea turtle-fishery interactions: Case study of East Coast Peninsular Malaysia.

[b22-tlsr-35-2-51] Zare R, Vaghefi ME, Kamel SJ (2012). Nest location and clutch success of the hawksbill sea turtle (*Eretmochelys imbricata*) at Shivdar Island, Iran. Chelonian Conservation and Biology.

